# Spatial transcriptomic analysis reveals lack of response to PD-1 blockade in recurrent glioblastoma

**DOI:** 10.1007/s00401-025-02937-9

**Published:** 2025-09-17

**Authors:** Sara Blaabjerg Artzi, Marc Nihøj Klausen, Dylan Scott Lykke Harwood, Signe Regner Michaelsen, Simone Bendix Maarup, Alessio Locallo, Vincent Fougner, Nicolai Schou Bager, Nadine Margaretha Hammouda, Dorte Schou Nørøxe, Benedikte Hasselbalch, Ulrik Lassen, Joachim Weischenfeldt, Bjarne Winther Kristensen

**Affiliations:** 1 Department of Clinical Medicine and Biotech Research and Innovation Centre (BRIC), University of Copenhagen, Copenhagen, Denmark; 2 Department of Pathology, Rigshospitalet, The Bartholin Institute, Copenhagen University Hospital, Copenhagen, Denmark; 3 DCCC Brain Tumor Center, Rigshospitalet, Copenhagen University Hospital, Copenhagen, Denmark; 4 Department of Oncology, Rigshospitalet, Copenhagen University Hospital, Copenhagen, Denmark; 5 The Finsen Laboratory, Rigshospitalet, Copenhagen, Denmark

**Keywords:** Glioblastoma, Recurrence, Immunotherapy, Spatial transcriptomics, Tumor microenvironment

## Abstract

Immune checkpoint inhibitors have transformed treatment for several cancers, yet clinical trials of programmed cell death protein 1 (PD-1) blockade in glioblastoma (GBM) have consistently failed to show therapeutic benefit. While some studies have reported treatment-related transcriptional changes, particularly in T cells, findings remain limited and inconsistent. The aim of this study was to investigate changes in tumor cells and tumor-associated macrophages (TAMs) after PD-1 blockade in recurrent GBM using spatial transcriptomics. We performed Digital Spatial Profiling (GeoMx, NanoString) on FFPE tumor samples from 26 patients with matched primary and recurrent IDH-wildtype GBM, including 16 patients who received neoadjuvant nivolumab at recurrence. Tumor (SOX2⁺) and TAM (IBA1⁺) segments were selected for targeted spatial analysis. Following quality control and filtering, transcriptomic profiles were compared between nivolumab-treated and untreated recurrent tumors. PD-1 blockade did not induce detectable gene expression changes in either tumor cells or TAMs. There were no significant differences in global expression profiles or in more targeted analyses of malignant cell states, cell cycle activity, interferon signaling, or myeloid transcriptional programs. These results consistently indicate that neoadjuvant PD-1 blockade does not elicit measurable responses at the spatial transcriptomic level in tumor cells or TAMs in recurrent GBM. These findings align with the lack of clinical benefit observed in trials and highlight the need for alternative strategies to improve immunotherapy outcomes in GBM.

**Supplementary Information:**

The online version contains supplementary material available at 10.1007/s00401-025-02937-9.

## Introduction

Glioblastoma (GBM) is the most common and aggressive malignant primary brain tumor in adults. Known for its rapid growth and diffuse infiltration, GBM exhibits high resistance to all therapies, including the standard-of-care treatment of radiotherapy (RT) with concurrent and adjuvant temozolomide (TMZ) [49]. Despite these treatments, most patients experience tumor recurrence within a year, with no standardized treatment options available at that point [52]. Clinical outcomes have remained largely unchanged over the past two decades, and the major challenges that hinder progress include pronounced tumor heterogeneity, a highly immunosuppressive microenvironment, and substantial tumor plasticity. These challenges highlight the critical need for the development of more effective treatment strategies.

Immunotherapies, such as PD-1 monoclonal antibody blockade, have been highly successful in treating several cancers by enhancing anti-tumor immune responses [8, 50, 51]. This therapy disrupts the interaction between PD-1 and its ligand PD-L1, a pathway that tumors exploit to evade immune detection [33]. By blocking this interaction, PD-1 inhibitors restore T cell activity, enabling cytotoxic T cells to eliminate tumor cells. However, to date, PD-1 blockade has failed to demonstrate any substantial survival benefit in clinical trials for patients with GBM [19, 29, 38, 41, 44, 45], with the exception of a few selected patient cases [1, 10, 53].

In light of this limited clinical efficacy, questions remain about whether PD-1 blockade induces any meaningful changes in the GBM tumor microenvironment (TME). To address this, several recent studies have employed “window of opportunity” trial designs, in which patients receive neoadjuvant treatment prior to tumor resection, allowing the analysis of tumor tissue shortly after therapy.

Bulk transcriptomic studies using this approach have reported suppression of cell cycle genes and upregulation of T cell- and interferon-related gene expression following neoadjuvant PD-1 blockade [10, 34]. Some also observed increased immune infiltration, greater T cell receptor clonal diversity among tumor-infiltrating T cells, and elevated expression of chemokines involved in immune cell recruitment within the TME [46].

Complementing these findings, single-cell studies have offered more nuanced insights into immune responses following PD-1 blockade. A combined mass cytometry and single-cell RNA sequencing (scRNAseq) study described increased T cell infiltration and activation, expansion of progenitor exhausted CD8 + T cells, and interferon-driven changes in myeloid populations [25]. However, immunosuppressive tumor-associated macrophages (TAMs) remained prevalent and expressed ligands for alternative checkpoints such as CTLA-4 and TIGIT, suggesting that targeting these pathways may be necessary to overcome TAM-mediated suppression and improve anti-tumor immunity. By contrast, another study using mass cytometry to profile the immune landscape in a smaller GBM cohort reported only a modest IFN-γ–driven immune response and found no significant changes in immune cell composition, T cell infiltration, or suppression of immunosuppressive myeloid populations following PD-1 blockade [17]. Finally, a scRNA-seq study of eight patients treated with neoadjuvant PD-1 blockade reported activation of a “latent immune signature” in two cases, marked by increased expression of tumor-inhibitory ligands and signs of T cell activation [21].

Taken together, these differing results highlight the uncertainty surrounding the extent and nature of the immune response to PD-1 inhibition in GBM, particularly beyond T cells. While most studies have centered on T cell responses, their overall impact in GBM may be limited, as T cells are generally sparse—comprising only 1–2% of all cells, with cytotoxic CD8⁺ T cells representing a small fraction of that [15].

In contrast to the limited T cell presence, TAMs represent the dominant immune population in GBM [9]. These cells are highly heterogeneous and exhibit profoundly immunosuppressive functions [23, 48]. While PD-1 blockade is primarily thought to act through T cell activation, its effects on TAMs remain poorly understood. Notably, PD-1 has been shown to be expressed on the surface of both human and mouse TAMs in colon cancer, where it inhibits phagocytosis and suppresses anti-tumor immunity. In vivo, PD-1 blockade was shown to enhance macrophage function and reduce tumor growth in a macrophage-dependent manner, further supporting a direct role in regulating TAM activity [16].

Given these findings, it remains unclear whether PD-1 blockade significantly impacts TAM function or gene expression in GBM. Addressing this gap requires studies specifically designed to evaluate responses within cell populations. Recent technological developments now allow spatially resolved transcriptomic profiling of protein-coding genes within selected cell populations in situ, enabling detailed characterization of cells immediately adjacent to each other.

Here, we expand on existing findings by using spatial transcriptomics (GeoMx) to evaluate the effects of PD-1 blockade on tumor cells and TAMs—the two most abundant cell populations in GBM. We analyzed tumor tissue from 30 patients with recurrent GBM (26 with IDH-wildtype tumors). Of these, 20 received neoadjuvant nivolumab (anti-PD-1) as part of the CA209-9UP trial [47], an open-label, window-of-opportunity phase II study in which patients received a single dose of nivolumab prior to surgery, followed by continued treatment postoperatively. Prior analyses of this same patient cohort, based on flow cytometry analyses, provided evidence of T cell engagement and activation following PD-1 blockade. Building on these findings, we investigated whether such immune activity is accompanied by transcriptional changes in tumor cells and TAMs. The remaining 10 patients served as matched untreated controls. Tumor regions with high densities of SOX2⁺ tumor cells and IBA1⁺ TAMs were selected for spatial profiling, enabling a focused profiling of PD-1 blockade-associated gene expression patterns within these cell types. While we did not observe significant transcriptional changes in tumor cells or TAMs following PD-1 blockade, our findings suggest that the impact of neoadjuvant PD-1 monotherapy in recurrent GBM may be limited.

## Materials and methods

### Patient selection

Archived, formalin-fixed, paraffin-embedded (FFPE) tissue blocks were obtained from 20 patients enrolled in the CA209-9UP trial (NCT03890952), an open-label phase II clinical study evaluating the effects of combined nivolumab and bevacizumab treatment in recurrent GBM (see [47] for trial details). Of these, 18 patients were part of the “surgical arm” and received a neoadjuvant dose of nivolumab prior to surgery for recurrent GBM. Two additional patients from the “non-surgical arm” underwent long-term nivolumab treatment until surgical resection became feasible. This trial cohort provided an opportunity to directly analyze tissue exposed to immunotherapy. Tissue samples were unavailable for two patients in the surgical arm.

When possible, matched FFPE blocks from each patient’s primary tumor were also included, allowing for direct comparison between primary and recurrent tumors within the trial cohort. Additionally, we incorporated archived FFPE tissue from 10 GBM patients outside the trial who had matched primary and recurrent tumor samples and had received standard treatment without nivolumab. This cohort served as a control group, enabling a baseline comparison against immunotherapy-exposed tissue to assess treatment-specific effects on tumor progression and immune responses.

All patients in our study cohort had previously been diagnosed with GBM. However, based on the latest WHO classification of brain tumors [31], four patients in the immunotherapy-treated group would now be classified as astrocytoma, *IDH*-mutant, WHO grade 4. These cases were excluded from further analysis to maintain consistency with current classification criteria.

All patients in this study were diagnosed between 2015 and 2021 and received treatment at the Department of Neurosurgery and Department of Oncology at Copenhagen University Hospital, Rigshospitalet. All had completed standard-of-care treatment with RT and TMZ at initial diagnosis. Patients’ clinical characteristics are listed in Supplementary Table 1.

### Tissue evaluation and tissue microarray (TMA) construction

Diagnostic H&E slides were evaluated using a BX51 microscope (Olympus Corporation) and reviewed by a neuropathologist (B.W.K.) to identify regions with the highest tumor cell density. Using an automated microarrayer, the TMA Grand Master (3DHISTECH), 2.0 mm diameter cores were extracted from these areas, with up to four cores taken per tumor. Each recipient block contained up to 40 cores, and six blocks were constructed in total.

### Genomic data

Whole-exome and whole-genome sequencing (WES/WGS) was performed on tumor and matched blood DNA using PCR-free library preparation. Sequencing depth was ≥ 60 × for tumor and ≥ 30 × for germline DNA, with ≥ 95% of the genome covered at ≥ 10x. FastQ files were quality controlled using FastQC (v0.11.8) and aligned to the human reference genome (GRCh38 for WGS and hg19 for WES) using BWA MEM (v0.7.15) [27]. Preprocessing followed GATK best practices [3], including duplicate marking and base quality score recalibration (GATK v4.1.9.0). Coverage metrics were calculated using GATK tools and mosdepth (v0.3.1) [43], with final QC reviewed using MultiQC (v1.9) [13]. Sample pairing was verified using Somalier (v0.2.11) [42], and stromal contamination and ploidy were estimated using Sequenza (v3.0.0) [14].

Somatic single nucleotide variants (SVNs) were called using MuTect2[5] and Strelka (v2.9.10) [24], using panels of normals (1000g_pon) and the gnomAD germline resource. Variant calls were merged with BCFtools (v1.3.1) [11], and filtered for variant allele frequency (VAF ≥ 5%). Functional annotation was performed using Ensembl VEP (v99.0) [35]. Somatic copy-number alterations were called using Sequenza.

The oncoplot summarizing genomic alterations and clinical features was generated using ComplexHeatmap (v2.18.0) [20] in R.

### GeoMx DSP: slide preparation and processing

We followed the GeoMx DSP Manual Slide Preparation User Manual (MAN − 10,150) for the RNA Slide Preparation Protocol. FFPE TMA blocks were sectioned to 5 μm using a Thermo Scientific HM 355S automated microtome and mounted onto TOMO slides. Slides were baked at 60 °C for 2 h in a BINDER FD 23 oven, then deparaffinized and rehydrated. Heat-induced epitope retrieval was performed in 1xTris-based buffer for 20 min at 100 °C. For RNA target exposure, slides were incubated with Proteinase K solution (Thermo Fisher Scientific, #AM2548, 0.1 μg/ml for 15 min) and post-fixed as described in the User Manual. Slides were hybridized with the GeoMx Human Whole Transcriptome RNA probe set (NanoString Technologies) and incubated overnight (20 h) in a HybEZ II Oven at 37 °C (Advanced Cell Diagnostics, a Bio-Techne brand).

Morphology markers included SOX2 (polyclonal, Novus, 1:100; 60 min incubation; detected using Alexa Fluor 532-conjugated goat anti-rabbit secondary antibody, Invitrogen A11009, 1:900; 20 min incubation), IBA1 (clone: GA5, Millipore, 1:25; 60 min incubation), and GFAP (clone: 20A12.1, Novus, 1:2000; 60 min incubation), with nuclei labeled using Syto13 (1:50; 30 min incubation). Slides were processed using the GeoMx DSP instrument according to the GeoMx DSP Instrument User Manual (MAN − 10,152–01), including scanning, region of interest (ROI) selection, segmentation, and barcode collection.

To reduce sampling bias and better account for intratumoral heterogeneity, multiple ROIs were selected per core whenever possible. On average, two ROIs approximately 600 μm in diameter were placed within each TMA core, guided by SOX2, IBA1, and GFAP staining, along with adjacent H&E reference slides. In some ROIs, cells were segmented based on IBA1 and SOX2 expression, while in others, segmentation was based on IBA1 and GFAP. In addition, multiple TMA cores were sampled per tumor to ensure that the spatial transcriptomic data captured more than a single tumor region, thereby increasing the robustness of downstream comparisons. Each tumor was represented by an average of 3.3 ± 1.0 TMA cores, totaling 7.7 ± 1.4 ROIs per tumor, for a dataset comprising 449 ROIs and 708 areas of illumination (AOIs).

DNA barcode libraries were prepared using the GeoMx DSP NGS Readout User Manual (MAN − 10,153) and sequenced on a NovaSeq 6000 platform (Illumina). BCL files were converted to FASTQ using Illumina’s bcl2fastq v2.20.0 software, quality-checked with FastQC v0.11.8, and processed into digital count conversion (DCC) files using NanoString’s GeoMx NGS Pipeline v2.3.

### GeoMx data normalization and filtering

To address count-based bias in the GeoMx platform, we applied quantile normalization (preprocessCore v1.64.0)[7] as suggested by van Hijfte et al.[22] A batch effect, due to two experiment runs, was corrected with Combat (sva v3.50.0) [26]. Genes were selected based on expression variance or mean, exceeding two standard deviations above negative probe counts. Tumor purity and malignancy in SOX2^+^ segments were inferred based on copy number variants (CNVs), alongside the expression of malignant cell state signatures defined by Neftel et al. [39] and the absence of non-malignant markers [12]. CNVs were assessed using inferCNV (v1.18.1) [54]. We clustered SOX2^+^ AOIs by CNVs on chromosomes 7 and − 10 (GBM hallmarks) and used supervised clustering to filter SOX2^+^ AOIs without CNVs and with high scores of non-malignant cells. IBA1^+^ AOIs were filtered based on TAM signature Z-scores.

### GeoMx statistical analyses

To analyze differences in GeoMx gene expression between treatment groups (treated vs. non-treated) and tumor settings (primary vs. recurrent), a linear mixed-effects model was employed to analyze differences in expression between treatment groups (treated vs. non-treated) and tumor settings (primary vs. recurrent). Patient identifier was included as a random effect in the model. Pairwise comparisons between all combinations of treatment and tumor settings were conducted using estimated marginal means with Tukey's adjustment for multiple comparisons. Statistical tests with *p*-values < 0.05 were considered significant. Adjustments for multiple testing were made using false discovery rate correction.

Differential gene expression analysis on GeoMx data was performed with DESeq2 (v1.42.1) [32], aggregating AOIs by tumor. The model compared nivolumab-treated and untreated recurrent tumors, without including *patient* as a covariate since only one tumor condition was tested per patient.

Centered and scaled data were used to calculate aggregated Z-scores across malignant cell state signatures defined by Neftel et al. [39], as well as additional signatures from Nomura et al. [40] and Greenwald et al. [18], enabling comparisons between trial settings. In addition, interferon response scores and myeloid program scores were computed using gene signatures from Miller et al. [37] Malignant states and cell cycle activity were evaluated in SOX2^+^ tumor cell segments, while interferon response was assessed in both SOX2^+^ and IBA1^+^ segments, comparing immunotherapy-treated and untreated recurrent tumors as well as primary versus recurrent tumors. Myeloid programs were analyzed in IBA1^+^ TAM segments from recurrent tumors only, to investigate potential PD-1 blockade-associated shifts in myeloid phenotypes.

### PD-1 protein expression by immunohistochemistry

To assess PD-1 protein expression in tissue sections, chromogenic immunohistochemistry (IHC) was performed semi-automatically using the Discovery ULTRA platform (Ventana/Roche). FFPE TMA sections, neighboring those used for GeoMx spatial transcriptomics, were used for staining. Sections were first deparaffinized, followed by heat-induced epitope retrieval using tris-based cell conditioning buffer (pH 8–8,5) at 100 °C for a total of 32 min. Endogenous peroxidase activity was blocked using a peroxidase inhibitor for 8 min. A primary rabbit monoclonal anti-PD-1 antibody (clone EPR4877(2), Abcam) was applied at a 1:150 dilution and incubated for 2 h at room temperature. Signal amplification was achieved through tyramide signal amplification (TSA) using TSA reagents, H₂O₂, and anti-HQ HRP. Subsequently, HRP-conjugated secondary antibody incubation was performed for 20 min. Visualization of antibody binding was done by HRP-catalyzed conversion of 3,3'-diaminobenzidine (DAB) to a brown precipitate using the ChromoMap DAB detection kit with copper enhancement (Ventana/Roche). Finally, nuclei were counterstained with hematoxylin II and bluing reagent.

Stained slides were digitized using a NanoZoomer S360 slide scanner at 40 × magnification. Images were analyzed with QuPath software (version 0.6.0) [4], where ROIs were manually outlined on TMA sections, excluding areas with hemorrhage, necrosis, or tissue folds. Positive cell detection applied a threshold-based algorithm optimized for the DAB optical density (OD) signal, using a single intensity threshold of 0.3 on the mean DAB OD per cell to classify PD-1 positive cells. Nuclei were detected and cell boundaries were estimated by expanding nuclei by 2.0 µm to capture cytoplasmic staining. Cell size parameters excluded small artifacts, removing cells smaller than 20 μm^2^ from analysis. The percentage of PD-1 positive cells was calculated relative to total detected cells per ROI. These values were exported for statistical analysis in R. Paired Wilcoxon signed-rank tests were used to compare PD-1 expression between matched primary and recurrent tumors within the same patients. Unpaired Wilcoxon rank-sum tests were used to compare PD-1 expression in recurrent tumors from immunotherapy-treated patients versus controls.

## Results

### Study design and patient cohort

**Fig. 1:**
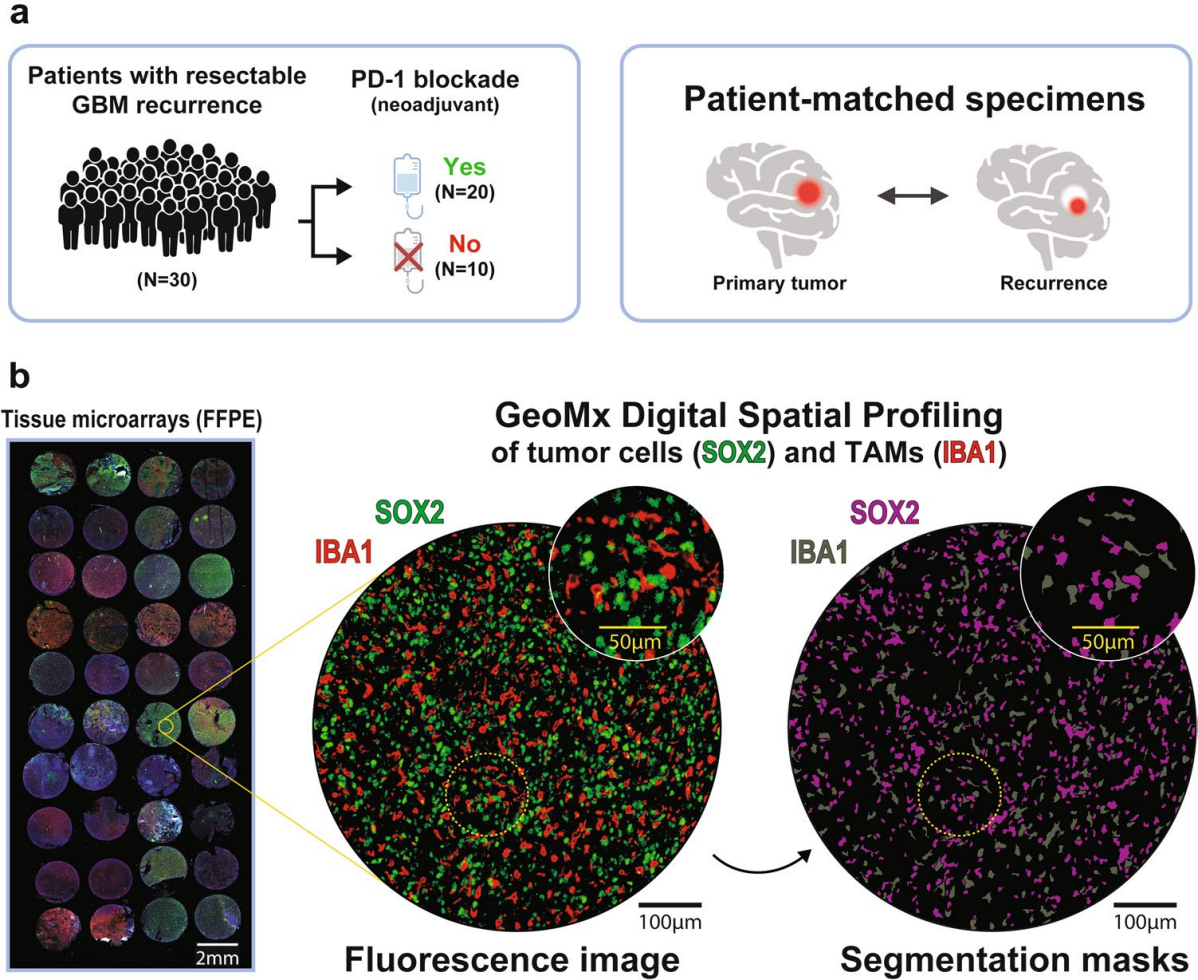
Spatial transcriptomic analysis of tumor cells and tumor-associated macrophages/microglia (TAMs) in recurrent GBM with or without neoadjuvant PD-1 blockade. **a** Overview of the tissue samples profiled in this study. **b** Representative fluorescence image from Digital Spatial Profiling (GeoMx) of an FFPE tissue microarray, showing SOX2^+^ tumor cells (green) and IBA1^+^ TAMs (red). Segmentation masks were applied to define tumor and immune regions based on morphology markers, enabling spatial transcriptomic profiling of distinct cell populations. Scale bars: black, 100 μm; yellow, 50 μm

To investigate the potential impact of PD-1 blockade on the recurrent GBM tumor and immune microenvironment, we used Digital Spatial Profiling (GeoMx, NanoString Technologies, Seattle, WA, USA) to spatially select and profile the transcriptomes of tumor cells (SOX2^+^) and tumor-associated macrophages (IBA1^+^) in FFPE tissue samples from matched primary and recurrent tumors, with immunotherapy administered at recurrence in a subset of patients (Fig. 1a).

Our study cohort included 30 patients with resectable GBM recurrence, 20 of whom were enrolled in the CA209-9UP trial, an open-label phase II study evaluating nivolumab, a PD-1 inhibitor, for recurrent GBM [47]. Although the trial did not demonstrate a benefit in either overall or progression-free survival, it remains unclear whether PD-1 blockade induces measurable biological responses in the tumor or immune microenvironment—insights that could help explain the lack of clinical efficacy and guide future strategies. Importantly, the authors were able to demonstrate intratumoral presence of nivolumab in a subset of treated patients, confirmed by flow cytometry and immunohistochemistry using an anti-IgG4 antibody—indicating that the drug had reached the tumor and was bound to intratumoral T cells.

Among the included immunotherapy-treated patients, 18 received a single 240 mg neoadjuvant dose of nivolumab 7 days prior to recurrence surgery, followed by continued treatment with nivolumab and bevacizumab after surgery. The remaining two patients underwent long-term nivolumab and bevacizumab treatment until surgical resection became feasible. Together, this provided an opportunity to assess anti-PD-1 treatment effects directly in the tumor tissue (see Methods for more details on patient inclusion and treatment protocols).

For the purpose of our study, we also included 10 control patients with recurrent GBM who had *not* received immunotherapy. These patients were selected based on various clinical parameters, including gender, age, performance score, and corticosteroid use prior to recurrence surgery, to achieve similar distributions across the two groups. Overall, steroid use was low or absent in most patients before surgery. Additionally, all patients were diagnosed and treated within a similar time period to minimize variability related to changes in clinical practice. Baseline clinical characteristics of the nivolumab-treated and control groups are summarized in Supplementary Table 2. In addition to profiling nivolumab-treated and untreated recurrences, our data set also included matching primary tumors from 27 of the 30 patients.

### GeoMx spatial profiling of tumor cells and TAMs

For spatial profiling, we constructed six FFPE tissue microarrays (TMAs), each containing up to 40 cores with a diameter of 2.0 mm (Fig. 1b). From each sample, up to four cores were selected from regions with the highest tumor cell density while excluding areas with large necroses or bleeding. This selection was verified by neuropathologist B.W.K.

We used the GeoMx Human Whole Transcriptome Atlas (WTA; 18,000 + genes) for spatial profiling. UV-photocleavable barcode-conjugated RNA in situ hybridization probes were applied to the FFPE TMA sections to capture and quantify mRNA counts from specific regions of interest (ROIs). The selection of ROIs was guided by immunofluorescence-based morphology marker staining for SOX2, IBA1, and GFAP, and ROIs were chosen based on the highest tumor cell density. SOX2, a transcription factor broadly expressed across stem-like and more differentiated glioma cells [6], was used to define tumor cell segments. IBA1, a marker specific to macrophages and microglia, was used to define TAM segments. Within each ROI, barcodes from the areas of illumination (AOIs) were cleaved, collected, and subsequently quantified through next-generation sequencing. To ensure minimal signal overlap between markers, barcodes from IBA1^+^ AOIs were collected first, followed by those from SOX2^+^ AOIs within the same ROI (see Methods for more details). On average, each AOI contained approximately 1,100 ± 842 SOX2⁺ cells and 245 ± 146 IBA1⁺ cells.

### Genomic profiling reveals hallmark GBM features and inter-patient heterogeneity

**Fig. 2:**
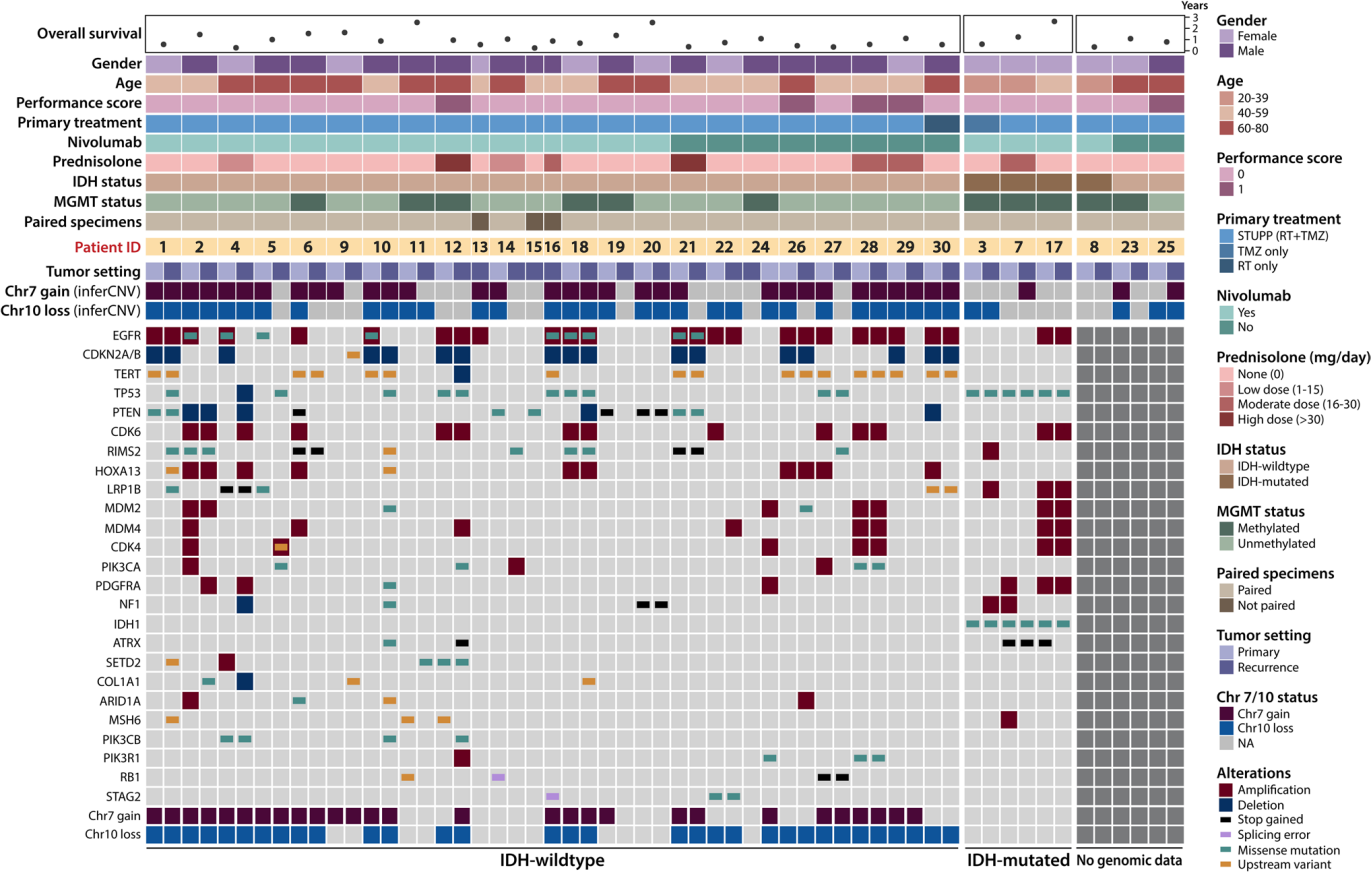
Oncoplot of clinical features and genomic alterations in recurrent GBM in our cohort, including both nivolumab-treated and control patients

To complement our spatial transcriptomic analyses, we performed genomic profiling to characterize molecular alterations across the cohort. This revealed substantial heterogeneity among patients, with a diverse range of alterations (Fig. 2). Gain of chromosome 7 and loss of chromosome 10, defining genomic features of GBM, were present in most IDH-wildtype tumors and were identified through whole-exome/genome sequencing and, as shown later, inferred from the transcriptional data generated in this study (see Methods). Alongside these broad alterations, *EGFR* amplification, *CDKN2A/B* deletion, and *TERT* promoter mutation were among the most common genomic changes observed. In some cases, although these alterations were detected in the primary tumor by at least one method, they could not be confirmed at recurrence—likely due to low tumor purity or DNA degradation.

While these findings collectively reaffirm the classification of these patients, they also highlight the extensive genomic heterogeneity of the disease, with diverse molecular alterations across patients. Furthermore, they hint at the challenges of studying recurrent tumors where tumor purity varies across samples, and the importance of identifying metrics to exclude samples not representative of tumor cells.

### Addressing low tumor purity in recurrent GBM samples

**Fig. 3:**
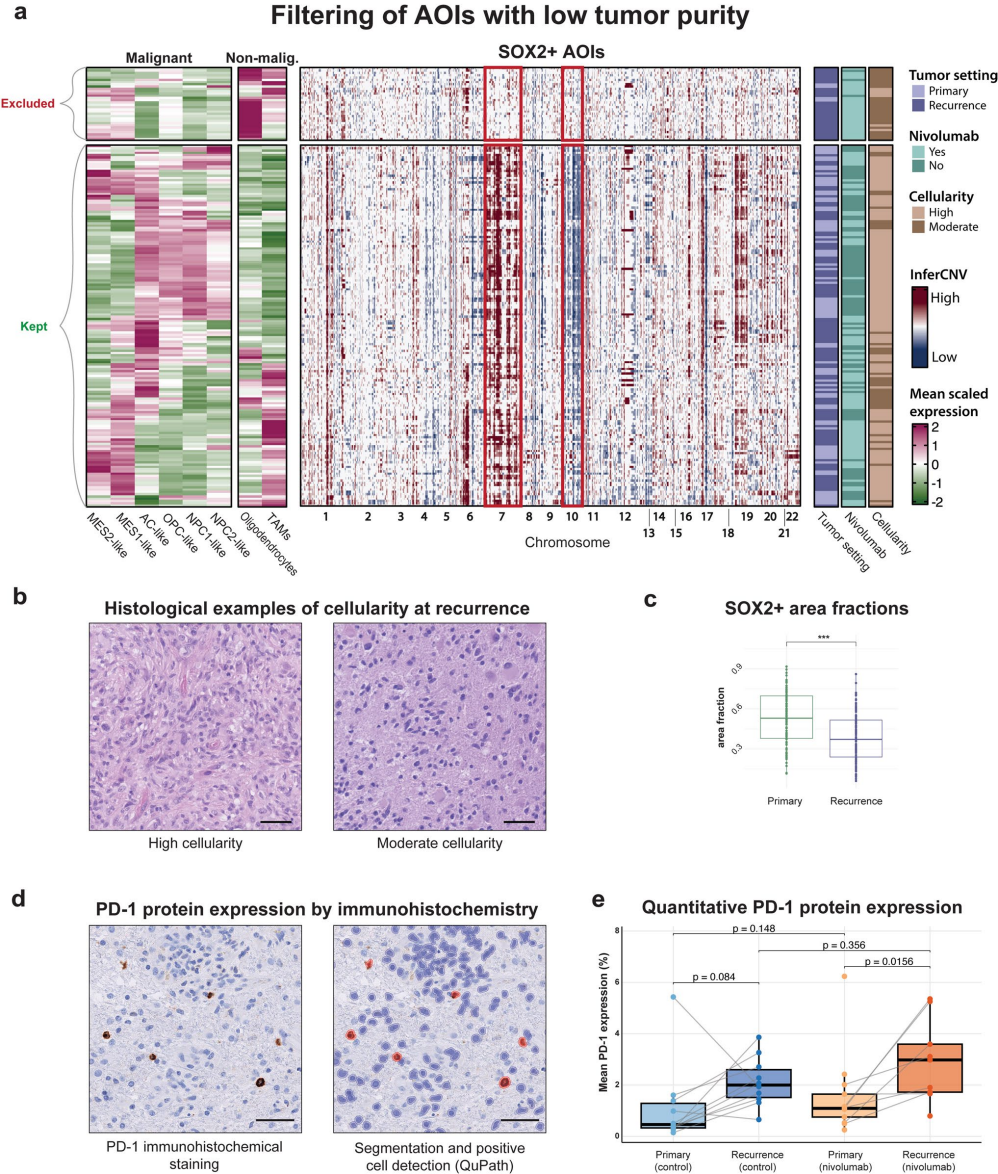
Tissue-level evaluation of SOX2^+^ regions and PD-1 expression in GBM samples. **a** Combined heatmap showing signature evaluations (left) and inferCNV results (center) for SOX2^+^ AOIs, annotated by tumor setting, nivolumab treatment, and cellularity. AOIs with low tumor purity, identified using the combined approach, are shown at the top of the heatmap and were excluded from the analysis. **b** Representative H&E images from recurrent tumor samples histologically annotated as having high (left) or moderate (right) cellularity. **c** Box plot comparing SOX2^+^ area fractions per ROI between primary and recurrent tumors. Each dot represents an individual ROI. *P*-value from linear mixed-effects model with patient as a random effect. **d** PD-1 protein expression by immunohistochemistry (left) and corresponding cell segmentation and positive cell detection using QuPath (right) in a representative tumor area. **e** Quantification of PD-1+ cells across groups based on mean positivity per tumor (aggregated across ROIs). Each dot represents one tumor; lines connect paired primary and recurrent tumors from the same patient. Exact p-values are shown. In **c**, the asterisk denotes a statistically significant difference (***p < 0.001). Scale bars: 50 μm (**b**, **d**)

As recurrent GBMs generally exhibit lower tumor density and a higher proportion of surrounding non-tumor cells, there is an increased risk of background contamination in GeoMx data due to the inherent limitations of its segmentation approach. To improve the reliability of our tumor cell comparisons, we implemented a two-step filtering strategy to identify and exclude SOX2^+^ AOIs with low tumor purity (Fig. 3a). First, we inferred copy number variations to detect hallmark GBM alterations—gain of chromosome 7 and loss of chromosome 10. Next, we evaluated each AOI’s gene expression profile against malignant (Neftel-defined) and selected non-malignant signatures, specifically those for oligodendrocytes and TAMs. AOIs that lacked CNVs and exhibited dominant non-malignant signatures (typically oligodendrocyte-like) were excluded from downstream analyses.

These excluded AOIs were mainly derived from recurrent tumors histologically annotated as having moderate cellularity (Fig. 3b), and overall, SOX2^+^ area fractions were significantly lower in recurrent tumors compared to primary tumors (Fig. 3c). Together, these observations are consistent with reduced tumor purity at recurrence and support the need for computational filtering to ensure the validity of comparative analyses. In total, four tumors from the nivolumab-treated group were excluded based on these criteria.

### Quantification of PD‑1 expression by immunohistochemistry

Using the same filtered tumor areas retained for transcriptomic analyses, we assessed PD-1 protein expression by chromogenic IHC and quantified PD-1^+^ cells using QuPath-based digital image analysis (Fig. 3d, e). Only TMA cores corresponding to high-purity SOX2^+^ tumor regions were included, to ensure consistency with the GeoMx filtering strategy. PD-1 positivity was defined as the percentage of positive cells per tumor, aggregated across ROIs. In the nivolumab-treated group (*n* = 7 pairs), PD-1 expression increased from a median of 1.11% (IQR: 0.59) in primary tumors to 3.11% (IQR: 2.61) in recurrent tumors (Wilcoxon signed-rank test, *p* = 0.0156). In the control group (*n* = 10 pairs), a similar but borderline significant increase was observed, from 0.46% (IQR: 0.96) to 2.00% (IQR: 1.08) (*p* = 0.084). Furthermore, comparison of nivolumab-treated recurrent tumors and untreated recurrences showed no significant difference in PD-1 expression (Wilcoxon rank-sum test, *p* = 0.356).

### Spatially resolved transcriptional analysis of tumor cells and TAMs reveals no significant impact of PD-1 blockade in recurrent GBM

To assess whether PD-1 blockade alters tumor cell or TAM transcriptional profiles, we performed spatially resolved analysis of SOX2^+^ and IBA1^+^ compartments.

**Fig. 4:**
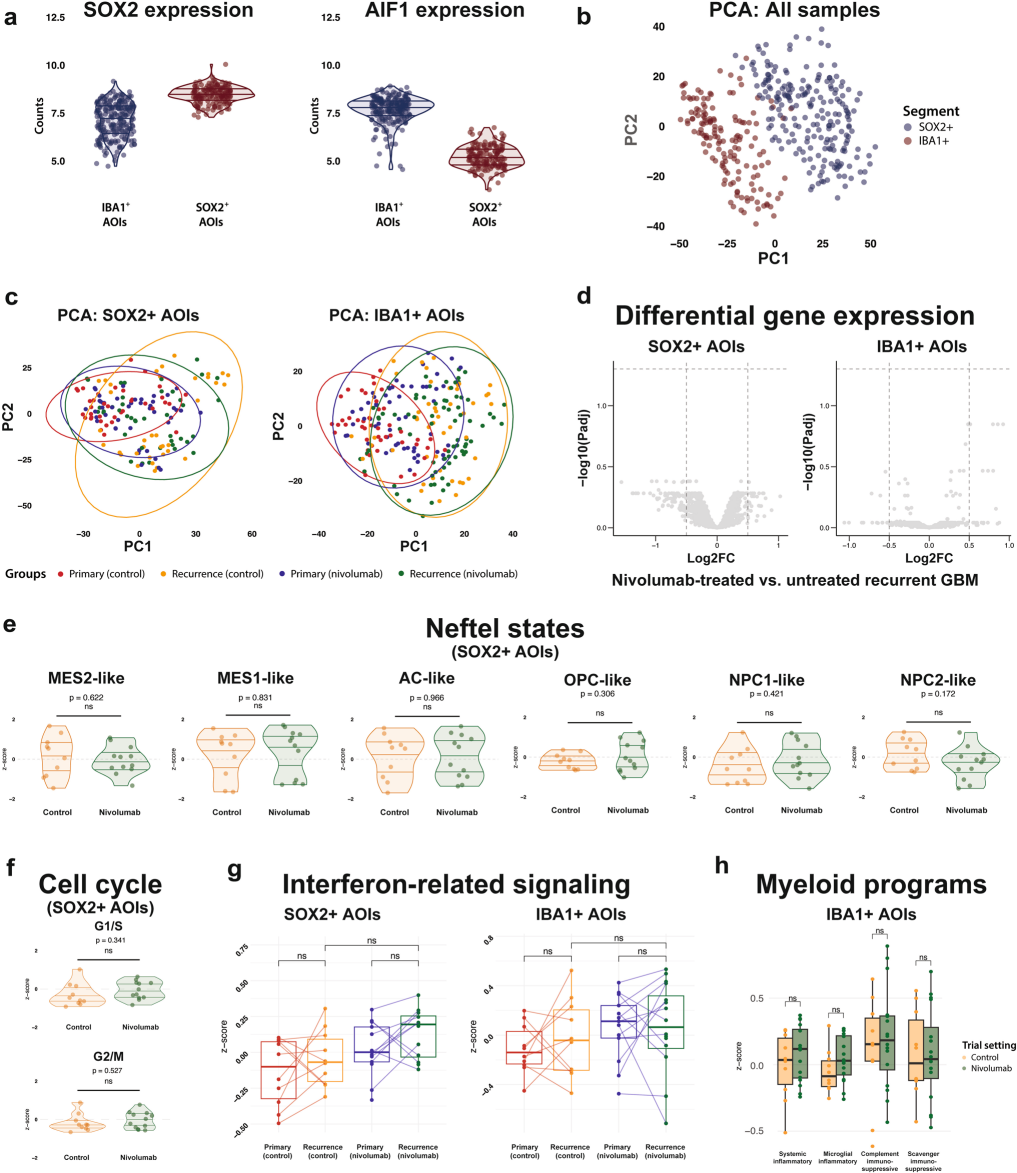
Spatial transcriptomic analysis of tumor cell (SOX2^+^) and TAM (IBA1^+^) segments following PD-1 blockade in recurrent GBM (post-filtering). **a** Violin plots showing *SOX2* and *AIF1* gene expression levels in transcriptomic data from both segments. **b** Principal component analysis (PCA) of all segments colored by segment type. **c** PCA plots of SOX2^+^ and IBA1^+^ segments, colored by tumor and trial setting. **d** Volcano plots showing differential gene expression between nivolumab-treated and untreated recurrent tumors in tumor cell and TAM segments. **e** Violin plots showing Neftel cell state scores in SOX2^+^ segments across trial settings. **f** Violin plots showing cell cycle signature scores (G1/S and G2/M) in SOX2^+^ segments across trial settings. **g** Box plots showing interferon signature scores in SOX2^+^ and IBA1^+^ segments across tumor and trial settings. **h** Box plot showing myeloid program scores in IBA1^+^ segments across trial settings. In panels **e**–**h**, each dot represents an individual tumor, with AOIs aggregated per tumor

After filtering, the expression of *SOX2* and *AIF1* (which encodes the protein IBA1) aligned with the expected segmentation, confirming distinct transcriptional profiles between tumor cells and TAMs (Fig. 4a). This was further supported by principal component analysis (PCA), which showed a clear separation between the two segments along PC1 (Fig. 4b).

To explore potential treatment effects, we next performed PCA separately for SOX2^+^ and IBA1^+^ AOIs across primary and recurrent tumors. This revealed no consistent clustering by treatment group, indicating a lack of major transcriptional shifts following PD-1 blockade (Fig. 4c). While a subset of recurrent control tumors showed distinct clustering in the SOX2^+^ compartment, this pattern was driven by elevated interferon signaling rather than treatment status.

Consistent with these observations, differential expression analysis revealed no significant differences between immunotherapy-treated and untreated recurrent tumors in either compartment (Fig. 4d). We next evaluated potential treatment-associated changes in malignant cell states and cell cycle progression. Using Neftel-defined transcriptional programs [39], we found no significant differences between treatment groups across the SOX2^+^ tumor cell segments (Fig. 4e). Similarly, G1/S and G2/M phase activity remained unchanged between groups (Fig. 4f), indicating no detectable impact of PD-1 blockade on cell cycle dynamics. To further explore potential treatment-associated shifts in tumor cell phenotypes, we also tested malignant cell state signatures derived from recent single-nucleus RNA sequencing [40] and spatial transcriptomic [18] studies (Supplementary Fig. 1). We again observed no significant differences between treatment groups across the SOX2 + segments, supporting the conclusion that PD-1 blockade did not measurably alter malignant cell states in recurrent GBM.

We also analyzed an interferon gene signature previously associated with immune activation in gliomas [37]. Although interferon scores were slightly higher in SOX2^+^ and IBA1^+^ segments from immunotherapy-treated recurrences, these differences were not statistically significant (Fig. 4g). Furthermore, some untreated recurrent tumors also showed high interferon activity, and many patients with elevated scores at recurrence already exhibited higher interferon signaling in their matched primary tumors. These findings suggest that interferon activity may be influenced more by patient-intrinsic factors than by PD-1 blockade.

Lastly, we also assessed expression of four recently defined myeloid programs from Miller et al. [37], including two immunosuppressive (*complement* and *scavenger*) and two inflammatory (*microglial* and *systemic*) signatures. These programs were also applied in their reanalysis of scRNA‑seq data from Mei et al. [36], where recurrent GBM patients received neoadjuvant PD‑1 blockade and were classified as responders (*n* = 7) or non‑responders (*n* = 5) based on MRI follow‑up, although this classification does not directly establish causality. Only the scavenger immunosuppressive program was associated with non-response to neoadjuvant PD-1 blockade, while no programs were associated with response. In our cohort, we did not observe any differences in expression of these programs between nivolumab‑treated and control tumors in any of the IBA1⁺ segments (Fig. 4h), suggesting that the lack of treatment effect we observed is not due to an upregulation of suppressive myeloid states.

Together, our spatial transcriptomic results do *not* support consistent transcriptional changes in tumor cells or TAMs in recurrent GBM following PD-1 blockade, suggesting that any potential effects are either absent, subtle, or highly variable across patients.

## Discussion

GBM remains one of the most challenging cancers to treat, with immunotherapy strategies, including PD-1 blockade, failing to demonstrate clinical benefit. Nonetheless, some prior studies have reported treatment-associated changes, although the evidence remains limited and somewhat inconsistent. In this study, we used spatial transcriptomics to investigate whether neoadjuvant PD-1 blockade induces detectable transcriptional changes in tumor cells and TAMs in recurrent GBM. Our aim was to characterize potential treatment-associated alterations in both tumor cells and the surrounding microenvironment, and to identify factors that may contribute to the observed lack of therapeutic response.

Although GBM is known for its profoundly immunosuppressive microenvironment, some previous studies have reported changes following PD-1 blockade—mainly within the immune compartment and particularly among T cells. However, in our study, we found no evidence of treatment-associated gene expression changes in TAMs or tumor cells following nivolumab treatment, suggesting that broader effects on the tumor microenvironment may be limited.

To explore this in depth, we first performed a global comparison of gene expression profiles between nivolumab-treated and untreated recurrent tumors. We then examined several additional features that could plausibly be influenced by PD-1 blockade or have been implicated in prior studies, including tumor cell cycle activity, transcriptional programs associated with malignant cell states, interferon-related signaling in both tumor cells and TAMs, and myeloid cell programs. Across all of these analyses, we observed no consistent or significant differences, indicating that PD-1 blockade does *not* induce detectable changes in the dominant tumor or immune cell populations in recurrent GBM—at least within an unselected patient population.

Our findings contrast with some earlier studies that reported transcriptional responses to neoadjuvant PD-1 blockade in GBM (summarized in Supplementary Table 3). Discrepancies between studies may reflect differences in methodology, tissue sampling, and patient inclusion criteria. Many previous studies relied on bulk expression profiling, included small or heterogeneous cohorts (often with IDH-mutant cases), or lacked appropriate untreated controls, which can make it difficult to isolate true treatment effects. Our study was designed to address these limitations by assessing the overall effect of treatment across a larger cohort using a direct comparison between immunotherapy-treated and untreated recurrent tumors. This comparison is essential for isolating treatment effects, as studies comparing primary and recurrent tumors—without untreated recurrence controls—may conflate immunotherapy responses with changes driven by standard treatment or disease progression.

By selecting tissue regions based on histology and segmenting tumor (SOX2^+^) and TAM (IBA1^+^) compartments, we reduced sampling variability across samples. This spatial approach also allowed us to exclude necrotic, non-tumor, or sparsely infiltrated areas, which may introduce noise and reduce the interpretability of transcriptional signals. In cases where tumor content was low or background contamination remained, we applied post-segmentation filtering to exclude such regions. Together, this two-step strategy—histology-guided preselection followed by stringent quality filtering—may explain why our results offer a more conservative and robust view of the local treatment response.

To further reduce biological heterogeneity, we restricted our analyses to IDH-wildtype tumors, acknowledging the distinct clinical and transcriptional features associated with IDH-mutant gliomas. This may also explain some of the variation observed across previous studies.

Despite these targeted strategies, we did not detect treatment-related effects in either tumor cells or TAMs—raising broader questions about *why* immune checkpoint blockade continues to fail in GBM. Recent reviews [2, 30] further emphasize the major barriers to effective immunotherapy in GBM. The lack of high tumor mutational burden, limited expression of PD-L1, and absence of robust interferon-signaling have all been associated with poor response to immune checkpoint inhibitors. Additionally, the profoundly immunosuppressive microenvironment—dominated by TAMs—and the physical constraints imposed by the blood–brain barrier may restrict immune cell trafficking and drug delivery.

A further challenge is the low abundance of T cells in GBM, with cytotoxic CD8⁺ T cells representing only a small fraction of the tumor. While our spatial transcriptomic analyses did not include T cells—due to their low abundance and segmentation limitations—we did assess PD-1 protein expression by chromogenic IHC. Although this method does not distinguish between cell types, PD-1 is primarily expressed by T cells, and many PD-1⁺ cells displayed lymphocyte-like morphology, suggesting they likely represent infiltrating T cells. These cells remain central to the proposed mechanism of PD-1 blockade, which is thought to promote anti-tumor immunity by reactivating exhausted T cells. The strongest evidence of PD-1 engagement in this setting comes from Skadborg et al. [47]. Although PD-1⁺ cell percentages increased at recurrence, they remained modest overall, and the sparse T cell infiltration in most GBM tumors likely limits the potential for broader immunologic effects. Indeed, if reactivated T cells were present in sufficient numbers to meaningfully impact the tumor, one might expect to detect downstream transcriptional changes in TAMs or tumor cells—such as increased interferon signaling or altered myeloid states—which we did not observe. This suggests that while PD-1 blockade may indeed activate T cells, as shown in the earlier study, such effects alone may not be sufficient to induce broader changes in tumor cells or TAMs. These factors collectively contribute to the resistance of GBM to PD-1 blockade and may help explain why even spatially targeted transcriptomic profiling reveals no detectable treatment effects in the tumor or macrophage compartments. These challenges highlight the need to better understand which patients, *if any*, might benefit from immunotherapy and whether specific tumor-intrinsic features contribute to resistance.

Importantly, our findings are consistent with the lack of clinical benefit observed across multiple trials of PD-1 blockade in GBM. Since no broadly applicable biomarker currently exists to guide patient selection, most trials to date have enrolled unselected patient populations, leading to substantial heterogeneity in treatment response. Although our results showed no consistent transcriptional changes following PD-1 blockade in either tumor cells or TAMs, they do not preclude the possibility that a subset of patients may still benefit from immunotherapy under specific biological conditions. However, identifying such predictive markers remains challenging. As discussed by Arrieta et al. [2], treatment response to immunotherapy in GBM is likely shaped by multiple factors, and no single biomarker has shown sufficient predictive value on its own. Instead, a more comprehensive approach—integrating clinical, genomic, and transcriptomic features—may be necessary to improve patient stratification. These results reinforce the need for biomarker-driven strategies, indicating that without patient selection, PD-1 blockade may have a limited effect on recurrent GBM.

Our study has a few important limitations to consider. First, while *Digital Spatial Profiling* enables targeted spatial analysis, it does not offer single-cell resolution, which may limit the detection of subtle transcriptional changes within rare or spatially localized cell populations. In addition, the sensitivity of GeoMx DSP is constrained by several factors, including the need to profile larger ROIs (typically ≥ 50 cells), the use of a limited number of probes per gene, and reliance on hybridization-based capture. These features, combined with potential RNA degradation in FFPE tissue, may reduce the ability to detect low-abundance transcripts [28]. While we cannot exclude the possibility that some focal or subtle changes were missed, the dominance of tumor cells and TAMs within each segment and the large number of profiled regions make it unlikely that widespread or biologically meaningful responses were overlooked. If treatment effects are this limited or subtle, their clinical relevance may also be questionable.

Another limitation is that most patients received only a single neoadjuvant dose of nivolumab prior to resection. It is possible that longer or combination treatment would be required to elicit broader effects. Although two patients in our cohort underwent extended treatment, this subgroup was too small for separate analysis. However, even within this limited timeframe, prior analyses from the same clinical trial showed that a single dose of nivolumab reached brain lesions, induced intratumoral T cell activation and proliferation, and upregulated additional immune checkpoints such as TIGIT, LAG-3, and TIM-3 within just seven days [47]. This suggests that early pharmacodynamic effects can occur in the T cell compartment, although it remains unclear whether these changes—or the absence of downstream transcriptional alterations in tumor cells or TAMs observed in our study—reliably predict longer-term treatment response or clinical benefit in GBM.

Finally, we chose to group samples based on treatment status alone to identify consistent gene expression differences in tumor cells and TAMs. However, it is possible that the PD-1 antibody triggered a checkpoint response only in a subset of patients, or that responses varied across different regions within individual tumors. Additional spatial-functional analyses will be needed to explore the potential co-occurrence of PD-1-mediated T cell activation, TAMs, and tumor cells.

Despite these limitations, our study represents the most comprehensive spatial transcriptomic analysis to date of tumor cells and TAMs following neoadjuvant PD-1 blockade in recurrent GBM. Despite a robust cohort, matched controls, and spatially guided profiling that minimized sampling bias, we found no consistent transcriptional changes in either compartment following treatment. These findings are in line with the lack of clinical benefit observed in trials of PD-1 monotherapy and reflect the continued difficulty of modulating the GBM microenvironment through immune checkpoint inhibition alone.

In conclusion, the absence of consistent changes in gene expression of tumor cells and TAMs suggests that the effects of PD-1 monotherapy in recurrent GBM are likely subtle and insufficient, highlighting the need for combination strategies and biomarker-driven patient selection in future trial designs. Our results demonstrate the value of cell type-specific, spatially resolved approaches for evaluating therapies in complex tumor ecosystems and provide a foundation for future translational studies and trial design in GBM.

## Supplementary Information

Below is the link to the electronic supplementary material.

Supplementary Fig. 1 Malignant cell state signatures from Nomura et al. and Greenwald et al. applied to SOX2^+^ tumor cell segments. (a) Violin plots showing malignant cell state scores based on Nomura et al. across SOX2^+^ segments from nivolumab-treated and untreated recurrent tumors. (b) Violin plots showing malignant state scores based on Greenwald et al. in the same samples. Each dot represents an individual tumor, with AOIs aggregated per tumor. Signature scores were centered and scaled prior to visualization. Corresponding p-values and FDR-adjusted values are indicated. (TIF 145996 KB)

Supplementary file2 (XLSX 8 KB)

Supplementary file3 (PDF 138 KB)

Supplementary file4 (PDF 154 KB)

## Data Availability

The GeoMx data generated in this study have been deposited in Zenodo and is accessible at 10.5281/zenodo.16839828. Any additional information regarding the data is available from the corresponding authors upon request.

## Code availability

The code used for data processing and analysis in this study is available upon request.

## References

[1] Arrieta VA, Chen AX, Kane JR, Kang SJ, Kassab C, Dmello C et al (2021) Erk1/2 phosphorylation predicts survival following anti-PD-1 immunotherapy in recurrent glioblastoma. Nat Cancer 2:1372–1386. 10.1038/s43018-021-00260-2 35121903 10.1038/s43018-021-00260-2 PMC8818262

[2] Arrieta VA, Dmello C, McGrail DJ, Brat DJ, Lee-Chang C, Heimberger AB et al (2023) Immune checkpoint blockade in glioblastoma: from tumor heterogeneity to personalized treatment. J Clin Invest. 10.1172/JCI163447 36647828 10.1172/JCI163447 PMC9843050

[3] van der Auwera G, O’Connor BD (2020) Genomics in the cloud: using Docker, GATK, and WDL in Terra, 1st edn. O’Reilly Media, Sebastopol

[4] Bankhead P, Loughrey MB, Fernández JA, Dombrowski Y, McArt DG, Dunne PD et al (2017) QuPath: Open source software for digital pathology image analysis. Sci Rep 7:16878. 10.1038/s41598-017-17204-5 29203879 10.1038/s41598-017-17204-5 PMC5715110

[5] Benjamin D, Sato T, Cibulskis K, Getz G, Stewart C, Lichtenstein L (2019) Calling somatic SNVs and indels with Mutect2. Broad Institute, Cambridge

[6] Berezovsky AD, Poisson LM, Cherba D, Webb CP, Transou AD, Lemke NW et al (2014) Sox2 promotes malignancy in glioblastoma by regulating plasticity and astrocytic differentiation. Neoplasia N Y N 16:193-206.e25. 10.1016/j.neo.2014.03.006 10.1016/j.neo.2014.03.006 PMC4094829 24726753

[7] Bolstad B (2023) preprocessCore: a collection of pre-processing functions. R package version 1.64.0. https://bioconductor.org/packages/preprocessCore. Accessed Apr 2024

[8] Brahmer JR, Tykodi SS, Chow LQM, Hwu W-J, Topalian SL, Hwu P et al (2012) Safety and activity of anti–PD-L1 antibody in patients with advanced cancer. N Engl J Med 366:2455–2465. 10.1056/NEJMoa1200694 22658128 10.1056/NEJMoa1200694 PMC3563263

[9] Charles NA, Holland EC, Gilbertson R, Glass R, Kettenmann H (2012) The brain tumor microenvironment. Glia 60:502–514. 10.1002/glia.21264 22379614 10.1002/glia.21264

[10] Cloughesy TF, Mochizuki AY, Orpilla JR, Hugo W, Lee AH, Davidson TB et al (2019) Neoadjuvant anti-PD-1 immunotherapy promotes a survival benefit with intratumoral and systemic immune responses in recurrent glioblastoma. Nat Med 25:477–486. 10.1038/s41591-018-0337-7 30742122 10.1038/s41591-018-0337-7 PMC6408961

[11] Danecek P, Bonfield JK, Liddle J, Marshall J, Ohan V, Pollard MO et al (2021) Twelve years of SAMtools and BCFtools. Gigascience 10:giab008. 10.1093/gigascience/giab008 33590861 10.1093/gigascience/giab008 PMC7931819

[12] De Falco A, Caruso F, Su X-D, Iavarone A, Ceccarelli M (2023) A variational algorithm to detect the clonal copy number substructure of tumors from scrna-seq data. Nat Commun 14:1074. 10.1038/s41467-023-36790-9 36841879 10.1038/s41467-023-36790-9 PMC9968345

[13] Ewels P, Magnusson M, Lundin S, Käller M (2016) MultiQC: summarize analysis results for multiple tools and samples in a single report. Bioinforma Oxf Engl 32:3047–3048. 10.1093/bioinformatics/btw354 10.1093/bioinformatics/btw354 PMC5039924 27312411

[14] Favero F, Joshi T, Marquard AM, Birkbak NJ, Krzystanek M, Li Q et al (2015) Sequenza: allele-specific copy number and mutation profiles from tumor sequencing data. Ann Oncol 26:64–70. 10.1093/annonc/mdu479 25319062 10.1093/annonc/mdu479 PMC4269342

[15] González-Tablas Pimenta M, Otero Á, Arandia Guzman DA, Pascual-Argente D, Ruíz Martín L, Sousa-Casasnovas P et al (2021) Tumor cell and immune cell profiles in primary human glioblastoma: Impact on patient outcome. Brain Pathol 31:365–380. 10.1111/bpa.12927 33314398 10.1111/bpa.12927 PMC8018082

[16] Gordon SR, Maute RL, Dulken BW, Hutter G, George BM, McCracken MN et al (2017) PD-1 expression by tumour-associated macrophages inhibits phagocytosis and tumour immunity. Nature 545:495–499. 10.1038/nature22396 28514441 10.1038/nature22396 PMC5931375

[17] Goswami S, Walle T, Cornish AE, Basu S, Anandhan S, Fernandez I et al (2020) Immune profiling of human tumors identifies CD73 as a combinatorial target in glioblastoma. Nat Med 26:39–46. 10.1038/s41591-019-0694-x 31873309 10.1038/s41591-019-0694-x PMC7182038

[18] Greenwald AC, Darnell NG, Hoefflin R, Simkin D, Mount CW, Gonzalez Castro LN et al (2024) Integrative spatial analysis reveals a multi-layered organization of glioblastoma. Cell 187:2485-2501.e26. 10.1016/j.cell.2024.03.029 38653236 10.1016/j.cell.2024.03.029 PMC11088502

[19] de Groot J, Penas-Prado M, Alfaro-Munoz K, Hunter K, Pei BL, O’Brien B et al (2020) Window-of-opportunity clinical trial of pembrolizumab in patients with recurrent glioblastoma reveals predominance of immune-suppressive macrophages. Neuro-Oncol 22:539–549. 10.1093/neuonc/noz185 31755915 10.1093/neuonc/noz185 PMC7158647

[20] Gu Z (2022) ComplexHeatmap: making complex heatmaps. R package version 2.18.0. https://bioconductor.org/packages/ComplexHeatmap

[21] Hendriksen JD, Locallo A, Maarup S, Debnath O, Ishaque N, Hasselbach B et al (2024) Immunotherapy drives mesenchymal tumor cell state shift and TME immune response in glioblastoma patients. Neuro-Oncol 26:1453–1466. 10.1093/neuonc/noae085 38695342 10.1093/neuonc/noae085 PMC11300009

[22] van Hijfte L, Geurts M, Vallentgoed WR, Eilers PHC, Sillevis Smitt PAE, Debets R et al (2023) Alternative normalization and analysis pipeline to address systematic bias in NanoString GeoMx digital spatial profiling data. iScience 26:105760. 10.1016/j.isci.2022.105760 36590163 10.1016/j.isci.2022.105760 PMC9800292

[23] Khan F, Pang L, Dunterman M, Lesniak MS, Heimberger AB, Chen P (2023) Macrophages and microglia in glioblastoma: heterogeneity, plasticity, and therapy. J Clin Invest 133:e163446. 10.1172/JCI163446 36594466 10.1172/JCI163446 PMC9797335

[24] Kim S, Scheffler K, Halpern AL, Bekritsky MA, Noh E, Källberg M et al (2018) Strelka2: fast and accurate calling of germline and somatic variants. Nat Methods 15:591–594. 10.1038/s41592-018-0051-x 30013048 10.1038/s41592-018-0051-x

[25] Lee AH, Sun L, Mochizuki AY, Reynoso JG, Orpilla J, Chow F et al (2021) Neoadjuvant PD-1 blockade induces T cell and cDC1 activation but fails to overcome the immunosuppressive tumor associated macrophages in recurrent glioblastoma. Nat Commun 12:6938. 10.1038/s41467-021-26940-2 34836966 10.1038/s41467-021-26940-2 PMC8626557

[26] Leek JT, Johnson WE, Parker HS (2023) sva: surrogate variable analysis. R package version 3.50.0. https://bioconductor.org/packages/sva. Accessed Apr 2024

[27] Li H, Durbin R (2009) Fast and accurate short read alignment with Burrows-Wheeler transform. Bioinforma Oxf Engl 25:1754–1760. 10.1093/bioinformatics/btp324 10.1093/bioinformatics/btp324 PMC2705234 19451168

[28] Lim HJ, Wang Y, Buzdin A, Li X (2025) A practical guide for choosing an optimal spatial transcriptomics technology from seven major commercially available options. BMC Genomics 26:47. 10.1186/s12864-025-11235-3 39833687 10.1186/s12864-025-11235-3 PMC11744898

[29] Lim M, Weller M, Idbaih A, Steinbach J, Finocchiaro G, Raval RR et al (2022) Phase III trial of chemoradiotherapy with temozolomide plus nivolumab or placebo for newly diagnosed glioblastoma with methylated MGMT promoter. Neuro-Oncol 24:1935–1949. 10.1093/neuonc/noac116 35511454 10.1093/neuonc/noac116 PMC9629431

[30] Liu Y, Zhou F, Ali H, Lathia JD, Chen P (2024) Immunotherapy for glioblastoma: current state, challenges, and future perspectives. Cell Mol Immunol 21:1354–1375. 10.1038/s41423-024-01226-x 39406966 10.1038/s41423-024-01226-x PMC11607068

[31] Louis DN, Perry A, Wesseling P, Brat DJ, Cree IA, Figarella-Branger D et al (2021) The 2021 WHO classification of tumors of the central nervous system: a summary. Neuro-oncol 23:1231–1251. 10.1093/neuonc/noab106 34185076 10.1093/neuonc/noab106 PMC8328013

[32] Love MI, Huber W, Anders S (2014) Moderated estimation of fold change and dispersion for RNA-seq data with DESeq2. Genome Biol 15:550. 10.1186/s13059-014-0550-8 25516281 10.1186/s13059-014-0550-8 PMC4302049

[33] Marasco M, Berteotti A, Weyershaeuser J, Thorausch N, Sikorska J, Krausze J et al (2020) Molecular mechanism of SHP2 activation by PD-1 stimulation. Sci Adv 6:eaay4458. 10.1126/sciadv.aay4458 32064351 10.1126/sciadv.aay4458 PMC6994217

[34] McFaline-Figueroa JR, Sun L, Youssef GC, Huang R, Li G, Kim J et al (2024) Neoadjuvant anti-PD1 immunotherapy for surgically accessible recurrent glioblastoma: clinical and molecular outcomes of a stage 2 single-arm expansion cohort. Nat Commun 15:10757. 10.1038/s41467-024-54326-7 39737895 10.1038/s41467-024-54326-7 PMC11685579

[35] McLaren W, Gil L, Hunt SE, Riat HS, Ritchie GRS, Thormann A et al (2016) The Ensembl Variant Effect Predictor. Genome Biol 17:122. 10.1186/s13059-016-0974-4 27268795 10.1186/s13059-016-0974-4 PMC4893825

[36] Mei Y, Wang X, Zhang J, Liu D, He J, Huang C et al (2023) Siglec-9 acts as an immune-checkpoint molecule on macrophages in glioblastoma, restricting T-cell priming and immunotherapy response. Nat Cancer 4:1273–1291. 10.1038/s43018-023-00598-9 37460871 10.1038/s43018-023-00598-9

[37] Miller TE, El Farran CA, Couturier CP, Chen Z, D’Antonio JP, Verga J et al (2025) Programs, origins and immunomodulatory functions of myeloid cells in glioma. Nature. 10.1038/s41586-025-08633-8 40011771 10.1038/s41586-025-08633-8 PMC12018266

[38] Nayak L, Molinaro AM, Peters K, Clarke JL, Jordan JT, de Groot J et al (2021) Randomized phase II and biomarker study of pembrolizumab plus bevacizumab versus pembrolizumab alone for patients with recurrent glioblastoma. Clin Cancer Res 27:1048–1057. 10.1158/1078-0432.CCR-20-2500 33199490 10.1158/1078-0432.CCR-20-2500 PMC8284901

[39] Neftel C, Laffy J, Filbin MG, Hara T, Shore ME, Rahme GJ et al (2019) An integrative model of cellular states, plasticity, and genetics for glioblastoma. Cell 178:835-849.e21. 10.1016/j.cell.2019.06.024 31327527 10.1016/j.cell.2019.06.024 PMC6703186

[40] Nomura M, Spitzer A, Johnson KC, Garofano L, Nehar-belaid D, Galili Darnell N et al (2025) The multilayered transcriptional architecture of glioblastoma ecosystems. Nat Genet. 10.1038/s41588-025-02167-5 40346361 10.1038/s41588-025-02167-5 PMC12081307

[41] Omuro A, Brandes AA, Carpentier AF, Idbaih A, Reardon DA, Cloughesy T et al (2023) Radiotherapy combined with nivolumab or temozolomide for newly diagnosed glioblastoma with unmethylated MGMT promoter: an international randomized phase III trial. Neuro-Oncol 25:123–134. 10.1093/neuonc/noac099 35419607 10.1093/neuonc/noac099 PMC9825306

[42] Pedersen BS, Bhetariya PJ, Brown J, Kravitz SN, Marth G, Jensen RL et al (2020) Somalier: rapid relatedness estimation for cancer and germline studies using efficient genome sketches. Genome Med 12:62. 10.1186/s13073-020-00761-2 32664994 10.1186/s13073-020-00761-2 PMC7362544

[43] Pedersen BS, Quinlan AR (2018) Mosdepth: quick coverage calculation for genomes and exomes. Bioinformatics 34:867–868. 10.1093/bioinformatics/btx699 29096012 10.1093/bioinformatics/btx699 PMC6030888

[44] Reardon DA, Brandes AA, Omuro A, Mulholland P, Lim M, Wick A et al (2020) Effect of nivolumab vs bevacizumab in patients with recurrent glioblastoma: the CheckMate 143 phase 3 randomized clinical trial. JAMA Oncol 6:1003–1010. 10.1001/jamaoncol.2020.1024 32437507 10.1001/jamaoncol.2020.1024 PMC7243167

[45] Reardon DA, Kim TM, Frenel J-S, Simonelli M, Lopez J, Subramaniam DS et al (2021) Treatment with pembrolizumab in programmed death ligand 1-positive recurrent glioblastoma: results from the multicohort phase 1 KEYNOTE-028 trial. Cancer 127:1620–1629. 10.1002/cncr.33378 33496357 10.1002/cncr.33378

[46] Schalper KA, Rodriguez-Ruiz ME, Diez-Valle R, López-Janeiro A, Porciuncula A, Idoate MA et al (2019) Neoadjuvant nivolumab modifies the tumor immune microenvironment in resectable glioblastoma. Nat Med 25:470–476. 10.1038/s41591-018-0339-5 30742120 10.1038/s41591-018-0339-5

[47] Skadborg SK, Maarup S, Draghi A, Borch A, Hendriksen S, Mundt F et al (2024) Nivolumab reaches brain lesions in patients with recurrent glioblastoma and induces T-cell activity and upregulation of checkpoint pathways. Cancer Immunol Res. 10.1158/2326-6066.CIR-23-0959 38885356 10.1158/2326-6066.CIR-23-0959 PMC11369628

[48] Sørensen MD, Dahlrot RH, Boldt HB, Hansen S, Kristensen BW (2018) Tumour-associated microglia/macrophages predict poor prognosis in high-grade gliomas and correlate with an aggressive tumour subtype. Neuropathol Appl Neurobiol 44:185–206. 10.1111/nan.12428 28767130 10.1111/nan.12428

[49] Stupp R, Mason WP, van den Bent MJ, Weller M, Fisher B, Taphoorn MJB et al (2005) Radiotherapy plus concomitant and adjuvant temozolomide for glioblastoma. N Engl J Med 352:987–996. 10.1056/NEJMoa043330 15758009 10.1056/NEJMoa043330

[50] Topalian SL, Hodi FS, Brahmer JR, Gettinger SN, Smith DC, McDermott DF et al (2012) Safety, activity, and immune correlates of anti–PD-1 antibody in cancer. N Engl J Med 366:2443–2454. 10.1056/NEJMoa1200690 22658127 10.1056/NEJMoa1200690 PMC3544539

[51] Topalian SL, Sznol M, McDermott DF, Kluger HM, Carvajal RD, Sharfman WH et al (2014) Survival, durable tumor remission, and long-term safety in patients with advanced melanoma receiving nivolumab. J Clin Oncol 32:1020–1030. 10.1200/JCO.2013.53.0105 24590637 10.1200/JCO.2013.53.0105 PMC4811023

[52] Wen PY, Weller M, Lee EQ, Alexander BM, Barnholtz-Sloan JS, Barthel FP et al (2020) Glioblastoma in adults: a Society for Neuro-Oncology (SNO) and European Society of Neuro-Oncology (EANO) consensus review on current management and future directions. Neuro-Oncol 22:1073–1113. 10.1093/neuonc/noaa106 32328653 10.1093/neuonc/noaa106 PMC7594557

[53] Zhao J, Chen AX, Gartrell RD, Silverman AM, Aparicio L, Chu T et al (2019) Immune and genomic correlates of response to anti-PD-1 immunotherapy in glioblastoma. Nat Med 25:462–469. 10.1038/s41591-019-0349-y 30742119 10.1038/s41591-019-0349-y PMC6810613

[54] (2025) inferCNV of the Trinity CTAT Project: inferring copy number variation from single-cell RNA-seq data. https://github.com/broadinstitute/inferCNV. Accessed Apr 2025

