# Supplementary material for: Spatial transcriptomic analysis reveals lack of response to PD-1 blockade in recurrent glioblastoma
Source: Acta Neuropathol. 2025 Sep 17;150(1):29. doi: 10.1007/s00401-025-02937-9 (PMC12443922; doi:10.1007/s00401-025-02937-9)
Supplement: Supplementary file 3 — Supplementary file3 (PDF 138 KB) [file 401_2025_2937_MOESM3_ESM.pdf]

Supplementary Table 2. Baseline clinical and treatment characteristics of the study cohort.

Characteristics	Total (N=26)	Nivolumab (N=16)	Control (N=10)
**Gender** - no. (%)			
Male	17 (65.4)	10 (62.5)	7 (70)
Female	9 (34.6)	6 (37.5)	3 (30)
**Age at diagnosis** (years), median (IQR)	61 (55.25, 67)	62.5 (56, 68)	56 (53.5, 64.25)
**MGMT promoter methylation** - no. (%)			
Unmethylated	19 (73.1)	11 (68.8)	8 (80)
Methylated	7 (26.9)	5 (31.2)	2 (20)
**Overall survival** (months), median (IQR)	10.44 (6.66, 13.14)	11.7 (7.83, 17.67)	7.8 (5.7, 12.09)
**Performance status (ECOG)** - no. (%)			
0	21 (80.8)	15 (93.8)	6 (60)
1	5 (19.2)	1 (6.2)	4 (40)
**Extent of primary resection** - no. (%)			
Total	21 (80.8)	13 (81.2)	8 (80)
Subtotal	5 (19.2)	3 (18.8)	2 (20)
**Corticosteriod dose at recurrence** (mg) - no. (%)			
0	19 (73.1)	12 (75)	7 (70.0)
12,5	2 (7.7)	2 (12.5)	0 (0.0)
20	1 (3.8)	1 (6.2)	0 (0.0)
25	2 (7.7)	0 (0.0)	2 (20.0)
50	2 (7.7)	1 (6.2)	1 (10.0)
**Primary treatment** - no. (%)			
Stupp	25 (96.2)	16 (100)	9 (90)
RT only	1 (3.8)	0 (0)	1 (10)
**Number of recurrences** - no. (%)			
1	20 (74.1)	13 (76.5)	7 (70)
2*	7 (25.9)	4 (23.5)	3 (30)
**Time between surgeries** (months), median (IQR)**	9.27 (7.02, 14.82)	10.7 (7.72, 16.87)	8.16 (6.31, 11.54)

* One patient was included with both their first and second recurrence (receiving long-term nivolumab treatment in between these two time points).
** This interval reflects the time from the preceding surgery (either primary resection or first recurrence) to the surgery at which the analyzed recurrent tissue was obtained.

Data are presented for all IDH-wildtype patients included in the study, shown overall and stratified by treatment group (neoadjuvant nivolumab vs. control). Values are given as counts and percentages or medians with interquartile ranges (IQR).
