# Supplementary material for: Spatial transcriptomic analysis reveals lack of response to PD-1 blockade in recurrent glioblastoma
Source: Acta Neuropathol. 2025 Sep 17;150(1):29. doi: 10.1007/s00401-025-02937-9 (PMC12443922; doi:10.1007/s00401-025-02937-9)
Supplement: Supplementary file 4 — Supplementary file4 (PDF 154 KB) [file 401_2025_2937_MOESM4_ESM.pdf]

Spatial transcriptomic analysis reveals lack of response to PD-1 blockade in recurrent glioblastoma (Acta Neuropathologica) - Sara Blaabjerg Artzi, Bjarne Winther Kristensen; University of Copenhagen & Copenhagen University Hospital, Rigshospitalet, Copenhagen, Denmark. Correspondence: sara.blaabjerg.artzi@regionh.dk; bjarne.winther.kristensen.01@regionh.dk

Supplementary Table 3. Summary of prior neoadjuvant ("window-of-opportunity") PD-1 blockade studies in recurrent glioblastoma.

Study	Main method(s)	Patient cohort (receiving PD-1 blockade)	IDH status	Control group	Main findings (associated with anti-PD-1 treatment)	Limitations
Hendriksen et al. (2024) [22]	scRNAseq	8 patients, treated with neoadjuvant nivolumab at recurrence	Mixed	Controls from external dataset (matched primary and recurrent tumors)	Identification of 'latent immune signature'/mesenchymal transition (two cases), increase of TAMs and proliferative and exhausted T cells in a subset of the treated patients	Limited sample size, no internal control group for single-cell analyses, inclusion of IDH-mutated patients
Skadborg et al. (2024) [48]	Flow cytometry	20 patients (surgical group), treated with neoadjuvant nivolumab at recurrence	Mixed	10 recurrent patients not receiving immunotherapy	Nivolumab reached brain lesions and saturated PD-1 on intratumoral T cells (as shown by anti-IgG4 staining detecting nivolumab bound to PD-1), evidence of increased T-cell activation and proliferation, and upregulation of checkpoint inhibitors (TIGIT, LAG-3, TIM-3, CTLA-4)	Limited exploration of other immune or tumor components (main findings center on T cell responses), inclusion of IDH-mutated patients
McFaline-Figueroa et al. (2024) [35]	RNA-seq	Follow-up study adding 25 patients to Cloughesy et al. (2019) cohort, treated with neoadjuvant pembrolizumab at recurrence	Mixed	Adjuvant-only treated controls (n=16) from the first study were used for comparison	Decrease in cell cycle-related genes in neoadjuvant group (not as pronounced in the expansion cohort), enrichment of T cell/interferon gene signatures in neoadjuvant group (this did not reach significance for the expanded neoadjuvant cohort)	Bulk-level gene expression profiling, no internal control group, inclusion of IDH-mutated patients
Lee et al. (2021) [26]	Mass cytometry, scRNAseq	20 patients treated with neoadjuvant pembrolizumab at recurrence	Not reported	22 recurrent patients not receiving immunotherapy	Increased infiltration of $CD8^+$ T cells, accompanied by interferon-related transcriptional changes in both T cells and myeloid populations, the immune microenvironment remained dominated by immunosuppressive TAMs expressing ligands for alternative checkpoints such as CTLA-4 and TIGIT	Tumor cells were not profiled (analyses were limited to immune cells), lack of patient-matched pre- and post-treatment samples, IDH mutation status was not clearly reported or controlled for
Goswami et al. (2020) [17]	Mass cytometry, targeted mRNA expression	5 patients treated with neoadjuvant pembrolizumab at recurrence	Not reported	7 primary tumor patients not receiving immunotherapy	Mass cytometry analyses: No significant changes in $CD4^+$ or $CD8^+$ T cell frequency, no significant changes in T-cell or myeloid cluster composition, CD73^hi immunosuppressive myeloid cells persisted after PD-1 blockade (not induced or eliminated by treatment). NanoString analyses: Modest increase in IFN-γ–responsive gene expression	Limited sample size, RNA analyses limited to pre-selected genes/bulk expression profiling, tumor cells were not profiled (analyses were limited to immune cells), IDH mutation status was not clearly reported or controlled for, comparisons were made between treated recurrent and untreated primary GBM
Cloughesy et al. (2019) [10]	RNA-seq, TCR-seq, quantitative mIF	16 patients in neoadjuvant group, treated with pembrolizumab	Mixed	16 recurrent patients treated with immunotherapy only in the adjuvant setting	Upregulation of T cell and interferon-γ related gene expression, downregulation of cell-cycle-related gene expression	Limited sample size, bulk-level gene expression profiling, inclusion of IDH-mutated patients
Schalper et al. (2019) [47]	Targeted mRNA expression, mIF, TCR-seq	30 patients (27 recurrent, 3 primary), treated with neoadjuvant nivolumab	Mixed	10 recurrent patients not receiving immunotherapy	Enhanced chemokine expression (e.g., CXCL10, CCL4, CCL3L1), increased immune cell infiltration - particularly activated CD8+ T cells (supported by flow cytometry but not mIF), augmented TCR clonal diversity among tumor-infiltrating lymphocytes	RNA analyses limited to pre-selected genes/bulk expression profiling, inclusion of both primary and recurrent tumors, use of corticosteroids during treatment (in 80% of patients), inclusion of IDH-mutated patients, several trial patients seem to have received additional treatment (in addition to Stupp), while this wasn't the case in the control group
